# FLASH Radiotherapy Using Single-Energy Proton PBS Transmission Beams for Hypofractionation Liver Cancer: Dose and Dose Rate Quantification

**DOI:** 10.3389/fonc.2021.813063

**Published:** 2022-01-13

**Authors:** Shouyi Wei, Haibo Lin, J. Isabelle Choi, Robert H. Press, Stanislav Lazarev, Rafi Kabarriti, Carla Hajj, Shaakir Hasan, Arpit M. Chhabra, Charles B. Simone, Minglei Kang

**Affiliations:** ^1^ New York Proton Center, New York, NY, United States; ^2^ Mount Sinai Hospital, New York, NY, United States; ^3^ Montefiore Medical Center, Bronx, NY, United States; ^4^ Memorial Sloan Kettering Cancer Center, New York, NY, United States

**Keywords:** FLASH radiotherapy, proton pencil beam scanning, dose rate, hypofractionation, liver cancer, stereotactic body radiation therapy, transmission proton beam, Bragg peak FLASH

## Abstract

**Purpose:**

This work aims to study the dose and ultra-high-dose rate characteristics of transmission proton pencil beam scanning (PBS) FLASH radiotherapy (RT) for hypofractionation liver cancer based on the parameters of a commercially available proton system operating under FLASH mode.

**Methods and Materials:**

An in-house treatment planning software (TPS) was developed to perform intensity-modulated proton therapy (IMPT) FLASH-RT planning. Single-energy transmission proton PBS plans of 4.5 Gy × 15 fractions were optimized for seven consecutive hepatocellular carcinoma patients, using 2 and 5 fields combined with 1) the minimum MU/spot chosen between 100 and 400, and minimum spot time (MST) of 2 ms, and 2) the minimum MU/spot of 100, and MST of 0.5 ms, based upon considerations in target uniformities, OAR dose constraints, and OAR FLASH dose rate coverage. Then, the 3D average dose rate distribution was calculated. The dose metrics for the mean dose of Liver-GTV and other major OARs were characterized to evaluate the dose quality for the different combinations of field numbers and minimum spot times compared to that of conventional IMPT plans. Dose rate quality was evaluated using 40 Gy/s volume coverage (V_40Gy/s_).

**Results:**

All plans achieved favorable and comparable target uniformities, and target uniformity improved as the number of fields increased. For OARs, no significant dose differences were observed between plans of different field numbers and the same MST. For plans using shorter MST and the same field numbers, better sparing was generally observed in most OARs and was statistically significant for the chest wall. However, the FLASH dose rate coverage V_40Gy/s_ was increased by 20% for 2-field plans compared to 5-field plans in most OARs with 2-ms MST, which was less evident in the 0.5-ms cases. For 2-field plans, dose metrics and V_40Gy/s_ of select OARs have large variations due to the beam angle selection and variable distances to the targets. The transmission plans generally yielded inferior dosimetric quality to the conventional IMPT plans.

**Conclusion:**

This is the first attempt to assess liver FLASH treatment planning and demonstrates that it is challenging for hypofractionation with smaller fractional doses (4.5 Gy/fraction). Using fewer fields can allow higher minimum MU/spot, resulting in higher OAR FLASH dose rate coverages while achieving similar plan quality compared to plans with more fields. Shorter MST can result in better plan quality and comparable or even better FLASH dose rate coverage.

## Introduction

FLASH radiotherapy (RT), characterized by an ultra-high-dose rate of >40 Gy/s as reported previously, has shown superior normal tissue protection and effective tumor control in many pioneering *in vivo* studies based on mice (1–5), minipig, cats (6), zebrafish (7), and first human treatment for a cutaneous lymphoma (8). While most of the abovementioned FLASH studies were based on electron beams (9), there have also been several investigations using photon beams (10) and increasingly using proton beams (11–17). The state-of-the-art proton beam delivery technique, pencil beam scanning (PBS), can precisely position each proton beamlet using scanning magnets, thereby providing outstanding target conformity and organ-at-risk (OAR) sparing. Moreover, proton beams are better able to treat tumors of all depths compared to electron beams. Furthermore, achieving high beam current from existing proton beam sources is much less challenging than photon beams. These features all make proton beams an attractive option for FLASH-RT (18).

In proton FLASH-RT, transmission proton beams have been the most favorable choice of delivery owing to the sufficiently high beam current achievable with existing clinical systems. Recent efforts have reported combining transmission proton PBS with FLASH-RT, to translate the technology from bench to preclinic experiments, in aspects including proton systems (11–15), treatment planning (18–23), and biological investigations (24, 25). Proton PBS FLASH treatment planning plays a crucial role just as conventional treatment planning, but it faces new and unique challenges as the dose rate considerations must be included when evaluating the plan quality. For proton PBS, currently, there is no widely accepted method to quantify the dose rate, despite multiple definitions proposed by different groups (19, 21, 22). There are several hypotheses, including oxygen depletion (26–29), immune response (30), and peroxyl radical recombination (31), that have been proposed to explain the FLASH sparing effect. Another important aspect for practical PBS FLASH treatment planning comes from the existing machine parameters (19, 32, 33), such as beam current, magnet scanning speed, and minimum monitor unit (MU) of a spot. These parameters may prevent OARs from reaching the FLASH dose rate coverage threshold during treatment planning. Zou et al. (33) discussed machine delivery limitations on phantom studies using an IBA proton system (Ion Beam Applications, Walloon Brabant, Belgium), but how such limitations affect the practical plan quality remains to be elucidated. Kang et al. studied the impact of minimum nozzle beam current on the plan quality and OAR FLASH coverage using the ProBeam system (Varian Medical Systems, Palo Alto, CA, USA), suggesting that the dose rate coverage will increase with the nozzle beam current and decrease with the field size (22).

Currently, several treatment planning studies have applied proton PBS FLASH beams to the head and neck (H&N) (19, 20, 34) and lung cancers (18, 22, 23, 35), with theoretical proton delivery parameters. van de Water et al. (19) discussed the feasibility of achieving transmission FLASH with existing and theoretical machine settings, especially the beam current, with the dose rate quantified using the dose-averaged dose rate (DADR). van Marlen et al. (18) applied transmission FLASH planning in lung and compared the plan quality to conventional non-FLASH plans using intensity-modulated proton therapy (IMPT). The liver is another promising treatment site suitable for hypofractionation and stereotactic body radiation therapy (SBRT) (36, 37) that potentially can benefit from the FLASH-RT sparing effect (38). Despite the existing H&N and lung studies showing promising treatment planning outcomes with transmission beams, FLASH-RT planning for the liver using hypofractionation has not been reported. The practical planning considerations such as dose, fractionation, and dose and dose rate constraints in OARs may bring additional challenges, especially when the fractionation dose is small and OAR dose constraints are strict. Also, how treatment planning strategies such as beam number and angles affect the plan quality, including FLASH dose rate coverage and other relevant technical parameters such as beam current and minimum MU/spot, has not yet been well defined (39). Such strategies, when used optimally, may improve the dose and dose rate quality suitable for FLASH-RT.

This work investigates liver-hypofractionated FLASH treatment planning with proton PBS transmission beams under practical and realistic planning settings. An inverse planning algorithm and a dose rate modeling were implemented using an in-house treatment planning system (TPS) to perform all transmission beam planning cases. The influence on the plan quality from the different number of fields and minimum spot time was evaluated using both dose and dose rate metrics. The dosimetric differences between conventional IMPT and transmission beams were also studied.

## Methods and Materials

### Dose Rate Modeling and Calculations

An in-house TPS, based on the matRad framework (40), was developed for inverse planning using transmission beams. The cyclotron beam current is variable in the Varian ProBeam system for different energy layers and automatically determined by the minimum MU per spot in the energy layer. Thus, an energy layer’s minimum MU/spot determines the deliverable dose rate (22). A single energy proton beam of 240 MeV was configurated in TPS. In a Varian ProBeam system, 1 MU corresponds to around 5.17 × 10^6^ protons for a 240-MeV beam (41). Denoting the minimum MU as MU_min_, beam current as I_beam_, minimum spot time (MST) as T_min_, and number of protons per MU as N_MU_, we will have the following relation in Eq. 1.


(1) $$MU_{\min}=I_{beam}*T_{\min}/N_{MU}$$

**Figure 1:**
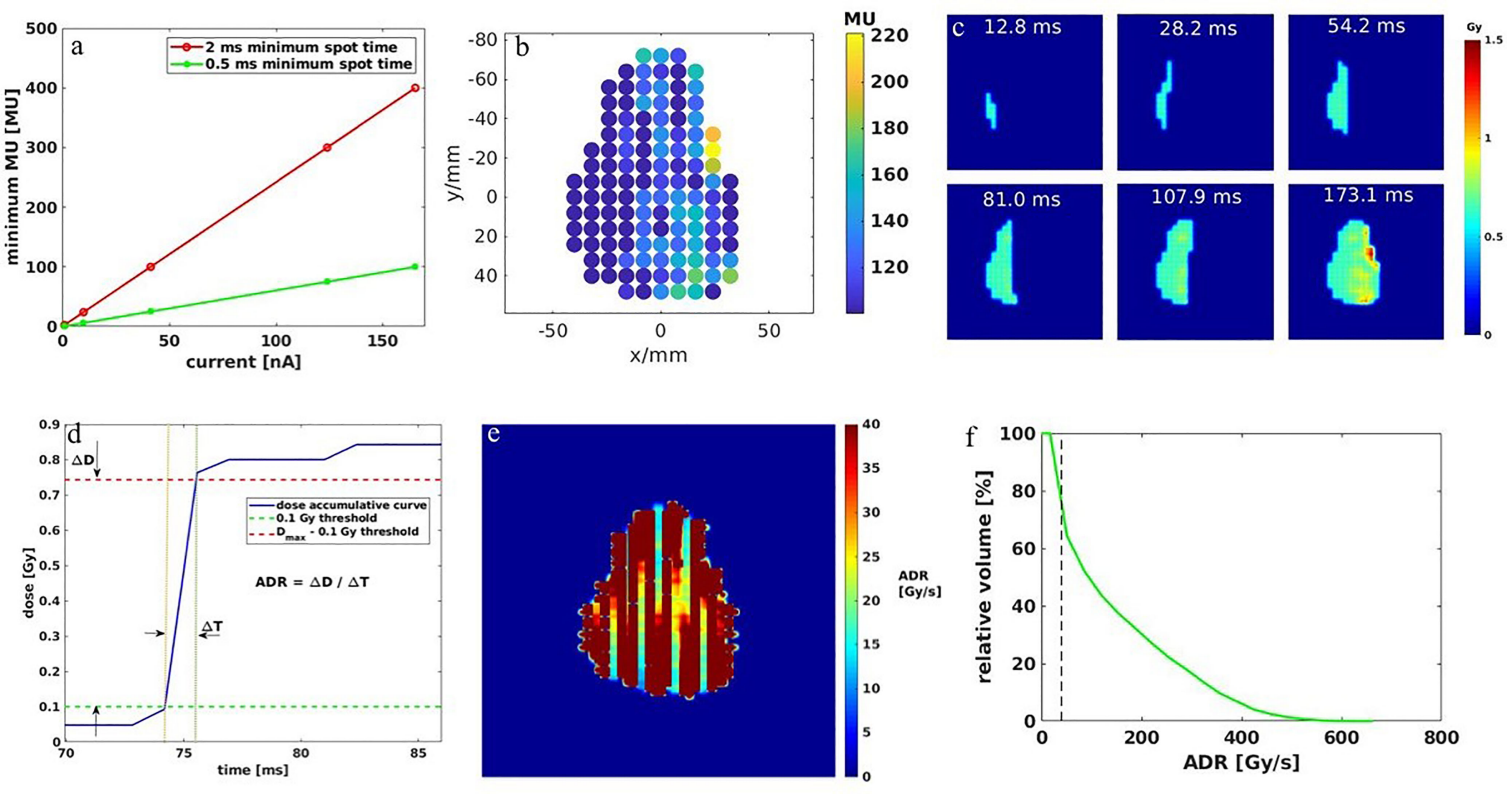
**(A)** Relationship between minimum MU and beam current for 2- and 0.5-ms MST. **(B)** A spot map with a minimum 100 MU/spot and 8-mm spot spacing. **(C)** Dose accumulation of the corresponding spot map in **(B)** over time. **(D)** Example of dose accumulation over time plot for one voxel inside a field. **(E)** The 2D dose rate map for the spot map is shown in **(B)**. **(F)** The dose rate volume histogram (DRVH) for the calculated dose rate map in **(E)**. Note the dashed line indicates the 40-Gy/s FLASH threshold.

Given a beam current of 165 nA, if the minimum spot time is 2 ms, the system delivers 2.07 × 10^9^ protons, approximately 400 MU, and for 0.5 ms, the delivered MU is 100. The linear relationships between the beam current and delivered minimum MU for the given MST are indicated in **Figure 1A**. An example of a spot map with 8-mm spot spacing is shown in **Figure 1B** with the minimum MU of 100 and MST of 0.5 ms. With the linkage between MU/spot and delivery time, we will be able to quantify the dose rate using the average dose rate (ADR), proposed by Folkerts et al. (21), where the dose rate is averaged over time for a given region of interest (ROI) in a scanning field. We used a 10-mm/ms scanning speed in the dose rate calculation.

The corresponding 2D dose accumulation changes with time are shown in **Figure 1C**. The dose accumulation plot of one voxel in the dose map is shown in **Figure 1D**, where the way the average dose rate is calculated is also indicated as the quotient between the defined accumulated dose and time duration. Then, the corresponding 2D dose rate map is shown in **Figure 1E**, where the dose rate distribution is scanning direction-dependent for a zigzag PBS pattern. Note that most voxels have a dose rate greater than 40 Gy/s, but still, some are below this threshold, especially in between the columns of the spots, which is reflected in the dose rate volume histogram (DRVH) as shown in **Figure 1F**.

### Hypofractionated FLASH Liver Treatment Planning

**Figure 2:**
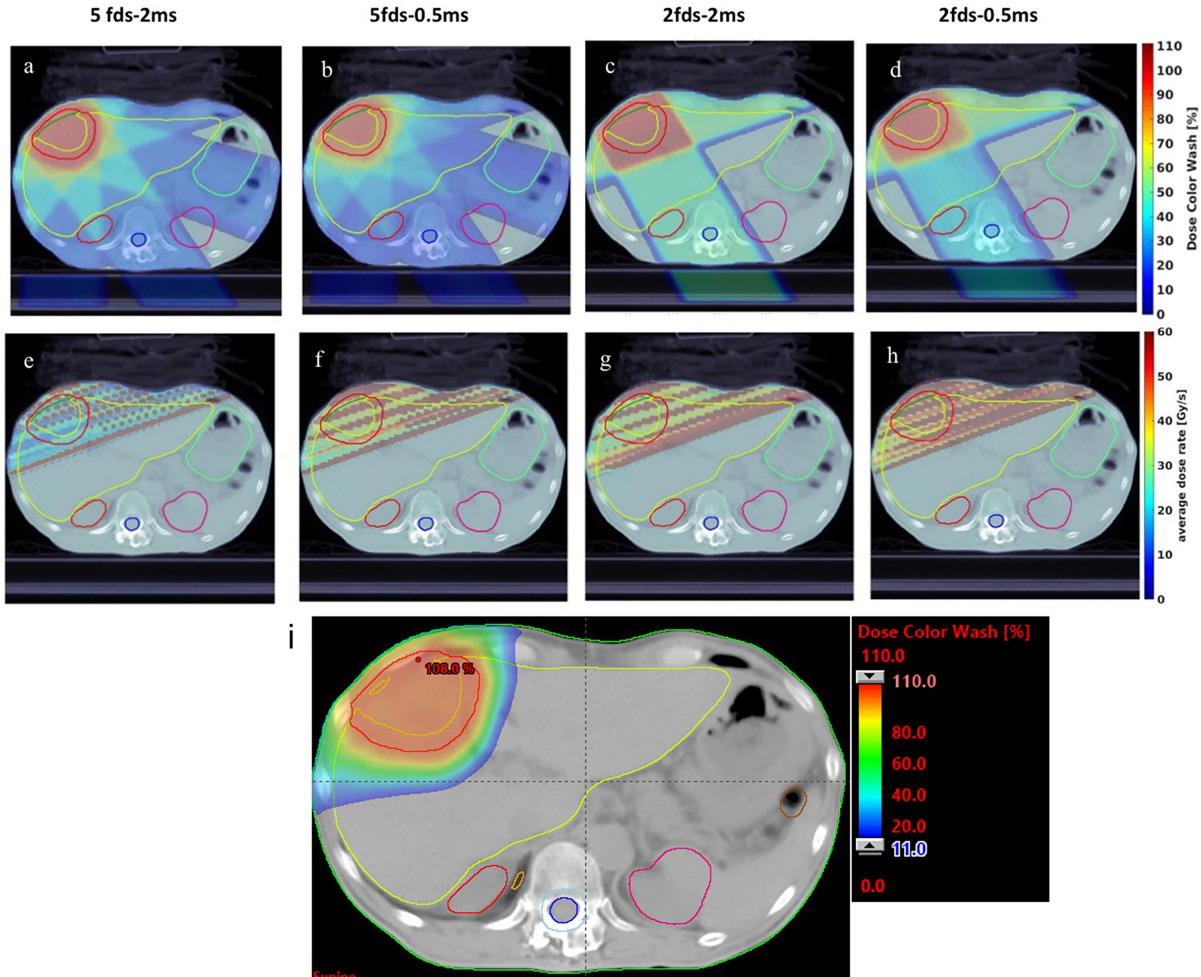
One example of the dose distribution for a single fraction: **(A)** 5 fields—2 ms MST, **(B)** 5 fields—0.5 ms, **(C)** 2 fields—2 ms, and **(D)** 2 fields—0.5 ms liver transmission plans, overlaid the CT images. The dose rate distribution for a single fraction: **(E)** 5 fields—2 ms MST, **(F)** 5 fields—0.5 ms, **(G)** 2 fields—2 ms, and **(H)** 2 fields—0.5 ms liver transmission plans, overlaid the CT images. **(I)** The 2D dose distribution of the SBRT plan for the same patient.

This study was conducted under institutional review board (IRB) approval. A cohort of 7 consecutive hepatocellular carcinoma patients previously treated with proton SBRT was replanned with single-energy transmission FLASH planning. All the transmission beams for the 7 cases were designed to ensure they were able to shoot through without the Bragg peaks being stopped inside the patients. The proton SBRT plans were optimized using either multiple-field optimization (MFO) or single-field optimization (SFO) with multiple energy layers, which deliver conformal doses to the target. A typical proton SBRT plan dose distribution is shown in **Figure 2I** as a reference for the FLASH transmission plans. The SBRT plan quality data were compared with the transmission plans to investigate the dosimetric characteristics of the FLASH plan.

All the dose was converted to relative biological effectiveness (RBE) dose using an RBE ratio of 1.1. The plans were prescribed to 67.5 Gy in 15 fractions. The internal clinical target volume (iCTV) was generated on an averaged CT by the union of CTVs on the corresponding 10-phase images of a 4D CT (42–44). The iCTV volumes ranged from 9.2 to 169.2 cm^3^ with a median value of 78.2 cm^3^. The beam angles in both 5-field and 2-field cases were chosen to ensure uniform target coverage while ensuring that OAR dose constraints could result in acceptable levels per the NRG Oncology GI003 protocol dose constraints (45). The machine and parameters for the different plan types are summarized in **Table 1**. For both 5 and 2 fields at 2 ms, the minimum MU/spot has to be adjusted ≤400, which corresponds to a beam current ≤165 nA to increase the target uniformity and meet OAR dose constraints. However, when the minimum spot time is reduced to 0.5 ms, all plans can use a much smaller minimum MU/spot of 100, equivalent to a beam current of 165 nA, to achieve good plan quality.

**Table 1:** Summary of machine and planning parameters for the investigated proton plans.

Plan type	Min MU	Min spot time (ms)	Field number	Dose per fraction/Gy
5fds–2ms	≤400	2	5	4.5
5fds–0.5ms	100	0.5	5	4.5
2fds–2ms	≤400	2	2	4.5
2fds–0.5ms	100	0.5	2	4.5

To evaluate the plan quality, we computed the NRG GI003 dose metrics from dose–volume histograms for different OARs after normalizing the 100% prescription dose to cover at least 95% of the target volume. As the distances between the target and OARs varied in the 7 cases, we chose to report the OARs that are most frequently irradiated, including liver-GTV, esophagus, spinal cord, stomach, kidney, chest wall, and heart. The RT structures and dose rate metrics studied and reported in this work are summarized in **Table 2**.

**Table 2:** Summary of dose metrics of the different number of fields and MST combinations for all 7 liver cancer patients compared to the conventional IMPT plans.

Structure	Dose metric	5 fields—2 ms	5 fields—0.5 ms	2 fields—2 ms	2 fields—0.5 ms	Conventional IMPT
iCTV	D_max_ (%)	115.2 (4.4)	113.6 (5.6)	117.6 (2.4)	114.1 (6.8)	107.1 (4.0)
Liver-GTV	D_mean_ (Gy)	18.1 (10.3) *^a^*	17.2 (9.8) *^a^*	17.9 (11.6) *^b^*	15.5 (10.5) *^b^*	12.4 (8.9)
Esophagus	D_0.5cc_ (Gy)	12.6 (5.6)	11.6 (6.3)	9.2 (14.8)	6.5 (11.4)	5.1 (8.7)
Spinal cord	D_0.5cc_ (Gy)	16.5 (7.9)	16.9 (8.5)	13.1 (11.6)	12.7 (10.5)	8.1 (10.5)
Stomach	D_0.5cc_ (Gy)	29.4 (19.4)	19.9 (6.2)	23.0 (31.2)	17.5 (21.4)	16.8 (25.5)
Kidney	V_18Gy_ (%)	12.6 (11.9)	11.8 (10.9)	19.3 (11.9)	15.8 (15.8)	5.6 (11.7)
Chest wall	D_2cc_ (Gy)	58.1 (21.0) *^a^*	55.2 (20.4) *^a^*	56.7 (20.5)	59.4 (17.6)	37.5 (14.3)
Heart	D_0.5cc_ (Gy)	31.8 (4.5)	30.9 (1.2)	11.5 (16.2)	7.9 (11.2)	8.5 (8.5)

The averages and standard deviations (in parentheses) are presented. Note: Heart D0.5cc only has two samples for each group, and t-tests were not applied.

a Indicates p < 0.05.

b Indicates p < 0.01, for paired Student t-test, between groups of the same field number and different minimum spot times.

The 3D dose rate of each field in the treatment plans was computed, then DRVHs for iCTV and each OAR were calculated to evaluate the FLASH dose rate of 40 Gy/s coverage. When using multiple fields to deliver FLASH treatment plans, the time spent between fields is much longer than each field time that is not included for dose rate calculation (22). Moreover, the voxels having non-zero doses of each field in a plan are included for calculating the DRVH. We introduce the metric of V_40Gy/s_, i.e., the percent volume covered by FLASH dose rate (>40 Gy/s), as the variable of merit. The dose and dose rate relationships were also studied for target and OARs. Six dose regions with 13.5-Gy dose intervals from 0 to 81 Gy were defined to quantitatively evaluate the dose rate statistical distribution vs. OAR doses.

## Results

### Dose Evaluation of the Liver Transmission Plans

**Figure 3:**
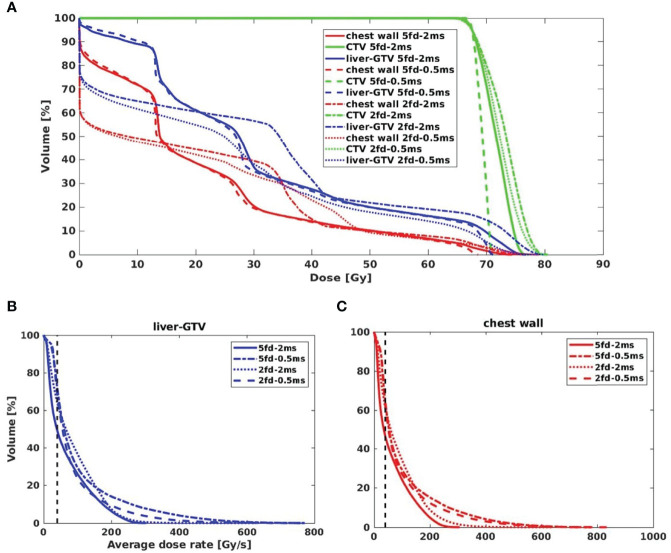
**(A)** The dose–volume histograms (DVH) of the iCTV and OARs for different fields and MST combinations. DRVH of **(B)** liver-GTV and **(C)** chest wall for four types of plans.

**Figures 2A–D** show an example of dose distributions of the 5-field and 2-field plans with 2 and 0.5 ms. It is evident that more fields distribute lower doses to larger body volumes, resulting in less target dose per field on average than plans using fewer fields. Although fewer fields are commonly associated with a higher exit normal tissue dose, the flexibility in angular arrangement makes it easier to spare particular OARs, such as the stomach and left kidney, in the representative case depicted in **Figures 2C, D**. As shown in **Figures 2B, C**, more fields and shorter MST appear superior to fewer fields and longer MST for target conformity and uniformity. For OAR doses, shorter MST allowed better modulation of the beam intensities and resulted in less normal tissue dose, such as the spinal cord dose of the 2-field plans in **Figure 2D**. The observations can be confirmed in the corresponding DVHs (**Figure 3A**). Compared with the transmission plans, the conventional SBRT plans using multiple energies (**Figure 2I**) can completely spare the OARs behind the target, such as the spinal cord and kidneys in this case.

For the 7 patients, as summarized in **Table 2**, more fields and shorter MST are consistently associated with smaller iCTV D_max_. As shown in **Table 2**, 5-field plans yield slightly less iCTV D_max_ than the 2-field plans at the same MSTs, and 0.5-ms MST plans also yield slightly less iCTV Dmax than the 2-ms MST ones when using the same number of fields. For the liver-GTV results, 0.5-ms MST leads to an average of 0.9 Gy (p < 0.05) and 2.4 Gy (p < 0.01) reduction compared to 2 ms MST. No statistical significance is identified between different combinations for the esophagus, spinal cord, and kidney. The 2-field plans have larger variability due to the selective OAR sparing in different patient cases. There is a significant D_2cc_ reduction of 2.9 Gy (p < 0.05) for the chest wall between the 0.5- and 2-ms MST in 5-field plans. Overall, iCTV and liver-GTV dose metrics have shown clear statistical characteristics between different combinations because all beams cover them in both 5-field and 2-field plans, which is not the case for other OARs. For the conventional IMPT plans, the iCTV D_max_ is > 5% better, and most of the OAR dose is reduced, for instance, by >3 Gy in liver D_mean_, >4 Gy in spinal cord D_0.5cc_, > 17 Gy in chest wall D_2cc_, and >6% in kidney V_18Gy_, compared with the transmission plans.

### Dose Rate Evaluation of the Liver Transmission Plans

**Figure 4:**
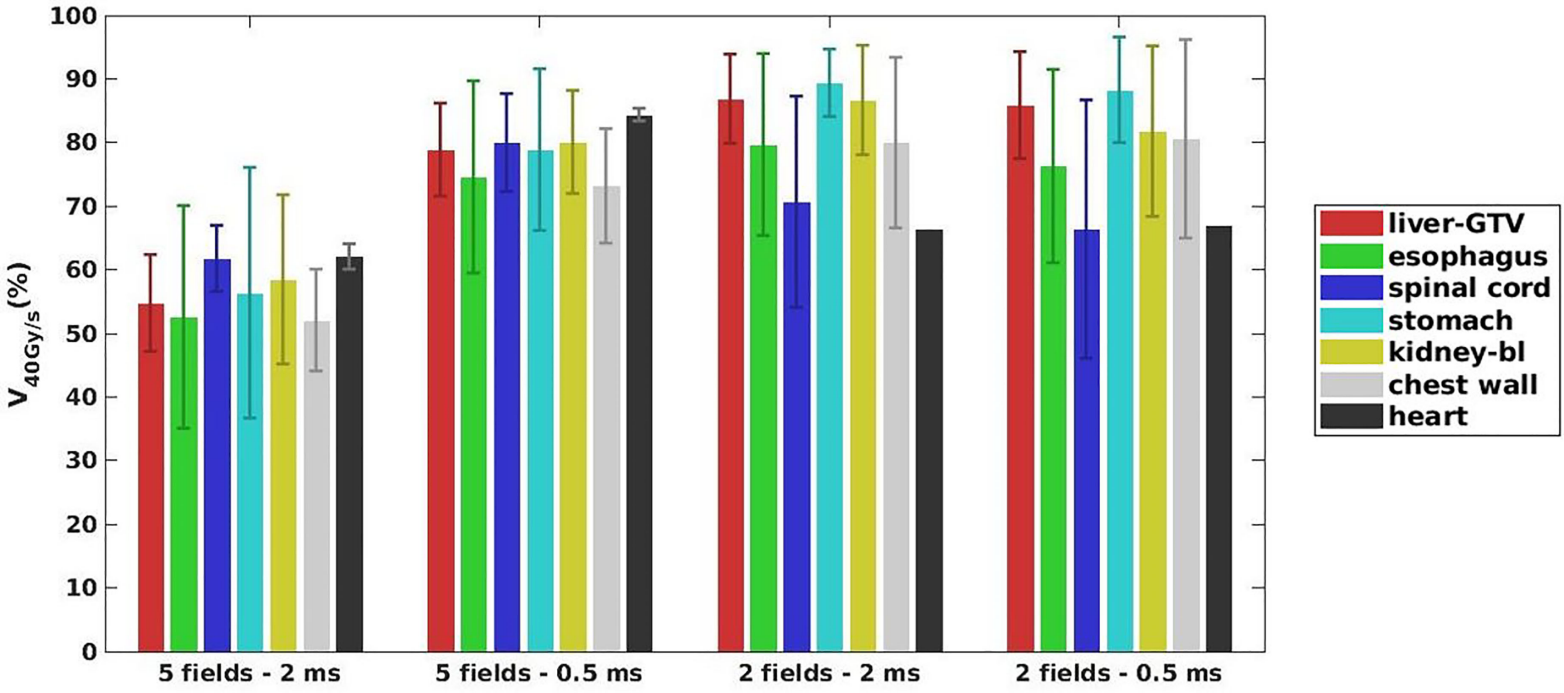
The average dose rate for all the 7 patients: OARs V40Gy/s distributions for different combinations of the number of fields and MST. The error bars represent the standard deviation from the mean value.

**Figure 5:**
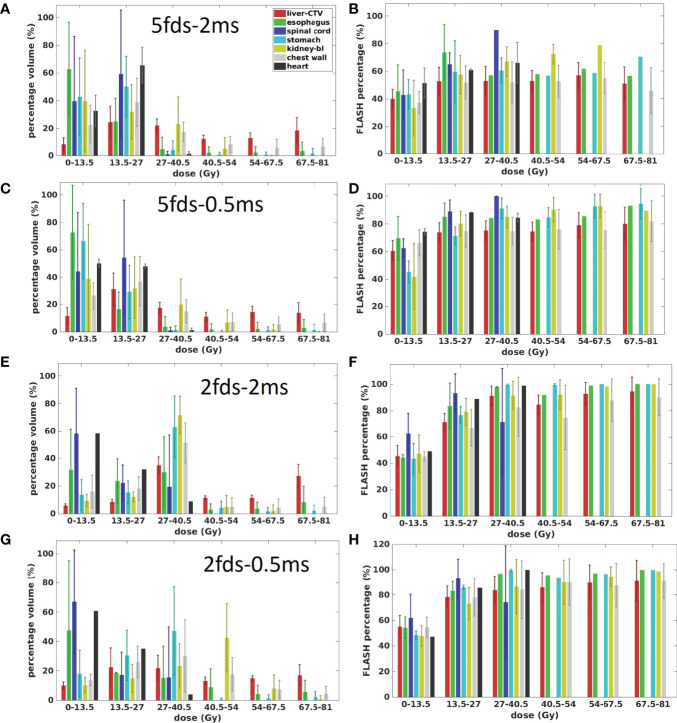
The dose and dose rate statistics of the four types of plans for all the 7 patients. **(A, C, E, G)** are the ratio of OARs’ volume in different dose regions for 5fds-2ms, 5fds-0.5ms, 2fds-2ms, and 2fds-0.5ms plans, respectively. **(B, D, F, H)** are the V_40Gy/s_ coverage of OARs and target changes under different dose levels for 5fds-2ms, 5fds-0.5ms, 2fds-2ms, and 2fds-0.5ms plans, respectively. The error bars represent the standard deviation from the mean value.

As shown in **Figures 2E–H**, for a typical liver case, 5fds-2ms plans (**Figure 2E**) result in the lowest dose rate coverage compared to the other generated plans. Plans with 2fds-0.5 ms (**Figure 2H**) have the highest dose rate coverage in liver-GTV for the same field, followed by the 2fds-2 ms (**Figure 2G**) and 5fds-0.5 ms (**Figure 2F**) ones. The differences in the liver-GTV and chest wall V_40Gy/s_ are also indicated in the DRVH (**Figures 3B, C**), where the lowest V_40Gy/s_ is observed with the 5fds-2ms plan, consistent with the dose rate distribution in **Figure 2E**. The 3D dose rate distribution is concentrated using a single DRVH curve to represent the voxel-based dose rate distribution. A single field’s 2D dose rate map displays the dose rate distribution variations among different combinations of fields and MST. The high-dose rate strips and low-dose rate valleys are observed, associated with the spot map distribution as seen in **Figures 2E–H**. **Figure 4** summarizes the V_40Gy/s_ FLASH dose rate coverage of OARs for all patients. It is evident that the 5fds-2ms MST plans have the lowest V_40Gy/s_ coverage in all cases, with over ~20% less than that of the 2fds-2ms MST plans for all OARs except for the spinal cord and the heart. The FLASH dose rate coverage differences are less evident between the 2- and 5-field plans with 0.5-ms MST, primarily within 10%, also bidirectional. For the liver-GTV, chest wall, and stomach, 2-field 0.5-ms MST plans yield slightly higher V_40Gy/s_. There are no apparent differences (less than 5%) between the 2fds-2ms MST and 2fds-0.5 ms MST plans.

### Dose and Dose Rate Relationships in Liver Transmission Plans

The OAR dose and dose rate relationships were inspected using the four types of plans in the transmission fields. **Figure 5** presents the dose and dose rate statistics of all four types of plans for all patients. As the target is located inside the liver, the liver-GTV received more dose than other OARs. While doses to OARs like the spinal cord, esophagus, stomach, kidney, chest wall, and heart are sensitive to the locations and beam arrangement, some of the OARs tend to have more low-dose portions and even can be completely spared if there was no beam passing through or toward them, as observed in all four types of plans (**Figures 5A, C, E, G**). In general, compared with others, the dose region of 0–13.5 Gy has the smallest FLASH dose rate coverage for all OARs in all four types of plans. For most OARs, the 5fds-2ms plans have the smallest FLASH dose rate coverage in the corresponding dose regions compared to all the other types of plans, which is consistent with the observation in **Figure 4**. As shown in the 2fd-2ms and 2fd-0.5ms cases (**Figures 5E, G**), for the spinal cord and heart, located farther away from the target, the lower-dose regions like 0–13.5 Gy are dominant, whereas, for the stomach, chest wall, kidney, and liver-GTV, the higher dose regions >27.5 Gy are dominant. As indicated in **Figures 5D, F**, the higher-dose regions correspond to a higher V_40Gy/s_ FLASH dose rate coverage. In comparison, the lower-dose regions correspond to a lower FLASH dose rate coverage. Nevertheless, as there are always low-dose regions like proton beam penumbras into OARs, it becomes less possible for OARs to reach 100% V_40Gy/_s dose rate coverage.

## Discussion

This study focuses on the FLASH-RT for liver hypofractionated treatment planning using transmission proton PBS beams. The dose rate model was implemented in an in-house TPS that incorporated the beam parameters of a commercial proton system under FLASH mode. The ADR calculation and evaluation methods were used to quantify the degree of expected FLASH effectiveness (“FLASHness”) in treatment planning.

Different groups have previously discussed the advantage of transmission beams in proton therapy (18, 22, 34). Treatment planning with transmission beams has also been studied in other sites such as lung and H&N. As the plateau region of proton beams is being used to deliver the prescribed dose to the target, the range uncertainty becomes less of a concern compared to the conventional Bragg peaks planning. Single-energy PBS transmission plans are also easier to implement without needing further beam modifiers in the beam path.

To our knowledge, this is the first study of applying transmission beams to the liver with practical dosimetric considerations. The relatively small fractionated dose leads to less flexibility in inverse planning, especially with relatively large minimum MU/spot required by FLASH dose rate coverage, limiting the achievable dose and dose rate performances. While achieving an ultra-high-dose rate would be technically easier to deliver with proton SBRT, since many lesions are not amenable to true ablative doses and since proton centers more typically deliver liver-directed radiotherapy using a hypofractionated approach rather than true SBRT approach in 5 or fewer fractions, we elected to similarly use hypofractionated FLASH to increase the generalizability and clinical usefulness of this analysis (46, 47).

This study adopted a more conservative PBS dose rate calculation method—ADR. As shown in other studies (22), DADR tends to result in higher values than ADR using the same beam current, as DADR specifies the dose-averaged instantaneous dose rate and ignores the time structures of spot deliveries in PBS (19). Currently, no studies have established which dose rate definitions are more relevant to the FLASH sparing effect. Also, it is still debatable in choosing which dose threshold to determine the dose and time window for ADR calculation. However, from the treatment planning point of view, we select ADR because if ADR can meet the FLASH coverage at a level, it can also be met by using DADR or other potential dose rate definitions.

The study indicates that to improve the FLASH coverage for OARs, we may reduce either the MST or the number of fields. Decreasing the number of fields can guarantee a higher minimum MU/spot for planning, which will be beneficial for achieving higher FLASH dose rate coverage; however, the fraction dose and OAR dose constraints can prevent using a very high minimum MU/spot for planning. As known, a smaller MU/spot will be helpful to ensure better dosimetry for OARs and targets. Therefore, the number of fields, dose rate coverage, plan quality, and minimum MU/spot are associated (39). When using fewer fields, the dose per field is increased, and the trade-off between FLASH dose rate coverage and dose constraints for OARs should be considered. **Figure 4** also indicates that compared to more fields using the same MST, an even lower FLASH coverage can occur to some OARs when the beam number is decreased, such as the spinal cord in the 2-field case. This is mainly because such OARs may happen to be at the penumbra of the beam, i.e., the low-dose region, which could be associated with a lower dose rate, as demonstrated in **Figure 5**. Applying a penumbra sharpening aperture (48) could be helpful to achieve both better OARs sparing and higher FLASH dose rate coverage. On the contrary, reducing the MST allows a lower minimum MU/spot in planning without degrading or even improving the FLASH coverage, as indicated in **Figures 4**, **5**. The MST can be optimized to accommodate both reasonably good dose rate and plan quality. If the MST can be set low enough, the number of fields may not be as important, as indicated in this liver planning case. However, when MST becomes a major obstacle in boosting the FLASH dose rate coverage, reducing the number of beams could be a useful strategy with carefully chosen beam angles that avoid major OARs while achieving acceptable plan quality. However, it may not be achievable for an extremely small MST due to hardware limits. The optimal and practical MST settings for the clinical FLASH delivery require further validations. The 2 and 0.5 ms investigated in this study are MSTs defined for the Varian ProBeam system, and other systems may have different mechanisms to determine the dose rate, which is not studied. We also note that the FLASH dose rate coverage may be affected by the OAR depths in the body, as the dose rate decreases when the depth increases, which warrants careful consideration in beam angular arrangement. Besides, giving lower minimum MU/spot constraints more flexibility, we may anticipate better plan quality, including target uniformity and OAR doses, as demonstrated in **Table 2**.

The doses to the OARs in the transmission plans are higher than SBRT plans on average due to the intrinsic limitation of the transmission plans that unavoidably deposit dose in normal tissues behind the target. Despite the transmission plans having higher OAR doses than the SBRT plans, the stringent dose constraints stated in the NRG Oncology GI003 are still met. As the FLASH spare effect for the normal tissue and OARs can potentially allow higher-dose tolerance than in the conventional dose rate radiation therapy, the slightly worse dosimetry of the transmission beams may not be a concern for the liver FLASH-RT. Till today, the biological mechanism of the FLASH dose rate has not been well characterized, and this planning study only provides a starting point to demonstrate the feasibilities of the application of transmission beams in liver FLASH treatment. More biological studies are expected to uncover the FLASH sparing effect for liver cancers.

In this study, we presume that proton RBE of FLASH irradiation in normal tissue is still 1.1, the same as the conventional dose rate proton irradiation. Although different RBEs for proton FLASH have been proposed (34), currently, there are no biological studies or experiments that have provided the measured RBE values. As we evaluate the “FLASHness” of each field, the impact from switching fields has not been considered, which requires further investigation relevant to fundamental FLASH mechanisms. Another potential problem for transmission FLASH-RT is that when patient size is large, the transmission beams cannot shoot through at certain beam angles. Also, transmission beams cannot spare the normal tissue at the distal edge of the target, which is validated by the comparison with conventional IMPT plans that place the Bragg peaks inside the targets. Although multiple-energy modulation as in conventional IMPT has not been available for FLASH-RT, using single-energy Bragg peak FLASH may provide a suitable solution to address the above issues, which we have evaluated by comparing the same dose metrics (23, 39). Some previous *in vivo* and *in vitro* experiments (49–51) suggest that the FLASH effect may be triggered when a particular dose threshold is met; what is a clinically feasible dose fractionation is still not clear yet for liver cancers. Further biological validation is needed, especially given the current lack of systematic clinical assessment with proton beams. This work evaluates the feasibility of achieving sufficient dose and dose rate quality based on the physical, planning, and machine parameters, and despite its limitations, it can provide reference to future FLASH planning under similar dosimetric requirements.

## Conclusion

Hypofractionated liver FLASH-RT planning was studied with single-energy proton transmission beams using an in-house TPS. We demonstrated that with a large MST, fewer-field plans will improve the dose rate quality marked by higher OARs’ FLASH coverages but with comparable dose quality to plans with a greater number of fields. With a small MST, more-field plans will have comparable dose rate quality and better dose quality compared to fewer-field plans. The inferior dose quality of transmission plans to conventional IMPT may justify the need to optimize treatment planning and dose delivery with comparable FLASH dose rate quality.

## Data Availability Statement

The original contributions presented in the study are included in the article/supplementary material. Further inquiries can be directed to the corresponding author.

## Ethics Statement

This study was conducted under Western institutional review board (IRB) approval. This is a retrospective study of previously implemented treatments and their dosimetry parameters, and there is no more than minimal risk to the subjects. Therefore, a waiver of informed consent was requested and approved by IRB.

## Author Contributions

Conceptualization, MK, SW, CS and HL. Methodology, MK, SW, CS, and HL. Software, SW. validation, MK, and SW. Formal analysis, SW. Investigation, MK, SW, and HL. Resources, JIC, CS, and HL. Data curation, MK and HL. Writing—original draft preparation, SW and MK. Writing—review and editing, SW, HL, JIC, RP, SL, RR, CH, SH, AC., CS. and MK. Visualization, MK and SW. Supervision, JIC, CS, and HL. Project administration, HL. Funding acquisition, JIC, CS, and HL. All authors contributed to the article and approved the submitted version.

## Conflict of Interest

The authors declare that the research was conducted in the absence of any commercial or financial relationships that could be construed as a potential conflict of interest.

## Publisher’s Note

All claims expressed in this article are solely those of the authors and do not necessarily represent those of their affiliated organizations, or those of the publisher, the editors and the reviewers. Any product that may be evaluated in this article, or claim that may be made by its manufacturer, is not guaranteed or endorsed by the publisher.
